# Tetra-μ-acetato-κ^4^ *O*:*O*′;κ^3^ *O*,*O*′:*O*;κ^3^ *O*:*O*,*O*′-bis­[(acetato-κ^2^ *O*,*O*′)(1,10-phenanthroline-κ^2^ *N*,*N*′)europium(III)]

**DOI:** 10.1107/S1600536810046131

**Published:** 2010-11-13

**Authors:** Rui-Qing Fan, Cun-Fa Sun, Yu-Lin Yang

**Affiliations:** a Department of Chemistry, Harbin Institute of Technology, Harbin 150001, People’s Republic of China

## Abstract

In the title centrosymmetric dinuclear complex, [Eu_2_(CH_3_CO_2_)_6_(C_12_H_8_N_2_)_2_], the Eu^III^ atom is nine-coordinated by two N atoms from a 1,10-phenanthroline ligand and seven O atoms from five acetate ligands (two bidentate, three monodentate). The crystal structure is stabilized by π–π stacking inter­actions between the pyridine and benzene rings of adjacent mol­ecules, with a centroid–centroid distance of 3.829 (2) Å.

## Related literature

For general background to lanthanide complexes based on nitro­gen-containing organic ligands, see: Lima *et al.* (2009 ▶); Prasad & Rajasekharan (2009 ▶); Xiang *et al.* (2009 ▶); Yang *et al.* (2009 ▶).
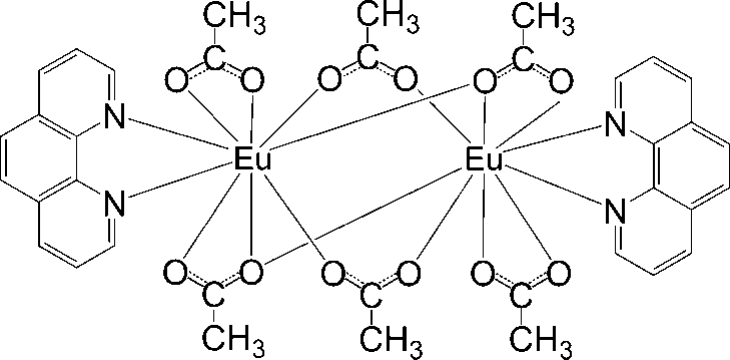


## Experimental

### 

#### Crystal data

[Eu_2_(C_2_H_3_O_2_)_6_(C_12_H_8_N_2_)_2_]
*M* *_r_* = 1018.59
Monoclinic, 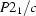

*a* = 9.7249 (19) Å
*b* = 23.670 (5) Å
*c* = 8.2984 (17) Å
β = 90.32 (3)°
*V* = 1910.2 (7) Å^3^
*Z* = 2
Mo *K*α radiation
μ = 3.32 mm^−1^
*T* = 293 K
0.20 × 0.10 × 0.08 mm

#### Data collection

Bruker APEX CCD diffractometer
Absorption correction: multi-scan (*SADABS*; Sheldrick, 1996 ▶) *T*_min_ = 0.559, *T*_max_ = 0.789
18210 measured reflections
4324 independent reflections
2668 reflections with *I* > 2σ(*I*)
*R*_int_ = 0.125

#### Refinement

*R*[*F* ^2^ > 2σ(*F* ^2^)] = 0.057
*wR*(*F* ^2^) = 0.101
*S* = 0.99
4324 reflections
244 parameters
H-atom parameters constrained
Δρ_max_ = 1.02 e Å^−3^
Δρ_min_ = −0.90 e Å^−3^

### 

Data collection: *SMART* (Bruker, 2007 ▶); cell refinement: *SAINT* (Bruker, 2007 ▶); data reduction: *SAINT*; program(s) used to solve structure: *SHELXS97* (Sheldrick, 2008 ▶); program(s) used to refine structure: *SHELXL97* (Sheldrick, 2008 ▶); molecular graphics: *SHELXTL* (Sheldrick, 2008 ▶) and *Mercury* (Macrae *et al.*, 2006 ▶); software used to prepare material for publication: *SHELXTL*.

## Supplementary Material

Crystal structure: contains datablocks global, I. DOI: 10.1107/S1600536810046131/hy2358sup1.cif

Structure factors: contains datablocks I. DOI: 10.1107/S1600536810046131/hy2358Isup2.hkl

Additional supplementary materials:  crystallographic information; 3D view; checkCIF report

## Figures and Tables

**Table 1:** Selected bond lengths (Å)

Eu1—O1	2.465 (5)
Eu1—O2	2.443 (5)
Eu1—O3	2.509 (5)
Eu1—O4	2.380 (5)
Eu1—O5^i^	2.387 (4)
Eu1—O6	2.372 (4)
Eu1—O6^i^	2.630 (5)
Eu1—N1	2.639 (6)
Eu1—N2	2.613 (6)

Symmetry code: (i) 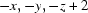.

**Fig. 1.**
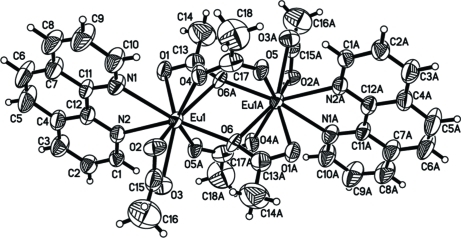
The molecular structure of the title compound. Displacement ellipsoids are drawn at the 50% probability level. [Symmetry code: (A) -x, -y, 2-z.]

**Fig. 2.**
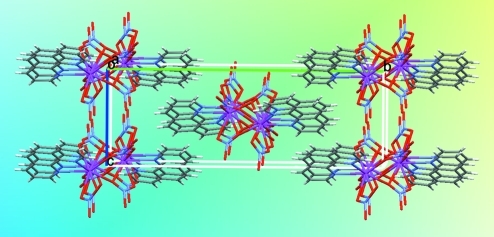
Packing diagram of the title compound along the a axis.

## supplementary crystallographic information

### Comment

Luminescent coordination compounds of lanthanide based on nitrogen-containing organic ligands have attracted intensive attention due to their potential applications in areas of sensor technologies and electro-luminescent devices (Xiang *et al.*, 2009; Yang *et al.*, 2009). In order to explore potential luminescent complexes of this type, a series of the lanthanide metal complexes with nitrogen-containing organic ligands have been studied (Lima *et al.*, 2009; Prasad & Rajasekharan, 2009). Here, we report the crystal structure of a dinuclear europium(III) complex with 1,10-phenanthroline ligand.

The title dinuclear complex consists of two Eu^III^ ions, two 1,10-phenanthroline ligands and six acetate anions (Fig. 1). The Eu atom is nine-coordinated by two N atoms from a phenanthroline ligand and seven O atoms from five acetate anions (Table 1).

There are three different linking fashions between acetates and Eu atoms. Each Eu atom is bonded to two O atoms from a chelating acetate (O2, O3), three O atoms from two chelating and bridging acetates (O1, O6, O6^i^) and two O atoms from two bridging acetates [O4, O5^i^; symmetry code: (i) -x, -y, 2-z]. Two nine-coordinated Eu atoms are linked by edge-sharing to form a dinuclear structure. It is noteworthy that π–π stacking interactions between adjacent phenanthroline ligands play a significant role in stabilizing the structure, with a centroid–centroid distance of 3.829 (2) Å (Fig. 2).

### Experimental

The title complex was prepared under mild conditions by allowing Eu(NO_3_)_3_ (0.043 g, 0.1 mmol) and 1,10-phenanthroline (0.059 g, 0.3 mmol) to react in a mixed solution of *N*,*N*-dimethylformamide (10 ml) and acetic acid (2.0 ml) at 338 K for 4 d. Colorless block crystals were obtained in a 47% yield based on Eu atom.

### Refinement

H atoms were positioned geometrically and refined as riding atoms, with C—H = 0.93 (aromatic) and 0.96 (methyl) Å and with *U*_iso_(H) = 1.2(1.5 for methyl)*U*_eq_(C). The highest residual electron density was found 0.92 Å from Eu1 and the deepest hole 0.95 Å from Eu1.

### Crystal data

**Table:**

[Eu_2_(C_2_H_3_O_2_)_6_(C_12_H_8_N_2_)_2_]	*F*(000) = 1000
*M**_r_* = 1018.59	*D*_x_ = 1.771 Mg m^−^^3^
Monoclinic, *P*2_1_/*c*	Mo *K*α radiation, λ = 0.71073 Å
Hall symbol: -P 2ybc	Cell parameters from 3465 reflections
*a* = 9.7249 (19) Å	θ = 3.3–27.5°
*b* = 23.670 (5) Å	µ = 3.32 mm^−^^1^
*c* = 8.2984 (17) Å	*T* = 293 K
β = 90.32 (3)°	Block, colorless
*V* = 1910.2 (7) Å^3^	0.20 × 0.10 × 0.08 mm
*Z* = 2	

### Data collection

**Table:**

Bruker APEX CCD diffractometer	4324 independent reflections
Radiation source: fine-focus sealed tube	2668 reflections with *I* > 2σ(*I*)
graphite	*R*_int_ = 0.125
φ and ω scans	θ_max_ = 27.5°, θ_min_ = 3.3°
Absorption correction: multi-scan (*SADABS*; Sheldrick, 1996)	*h* = −12→12
*T*_min_ = 0.559, *T*_max_ = 0.789	*k* = −30→30
18210 measured reflections	*l* = −9→10

### Refinement

**Table:**

Refinement on *F*^2^	Primary atom site location: structure-invariant direct methods
Least-squares matrix: full	Secondary atom site location: difference Fourier map
*R*[*F*^2^ > 2σ(*F*^2^)] = 0.057	Hydrogen site location: inferred from neighbouring sites
*wR*(*F*^2^) = 0.101	H-atom parameters constrained
*S* = 0.99	*w* = 1/[σ^2^(*F*_o_^2^) + (0.0299*P*)^2^] where *P* = (*F*_o_^2^ + 2*F*_c_^2^)/3
4324 reflections	(Δ/σ)_max_ < 0.001
244 parameters	Δρ_max_ = 1.02 e Å^−^^3^
0 restraints	Δρ_min_ = −0.90 e Å^−^^3^

### Fractional atomic coordinates and isotropic or equivalent isotropic displacement parameters (Å^2^)

**Table:**

	*x*	*y*	*z*	*U*_iso_*/*U*_eq_	
Eu1	0.13542 (4)	0.060026 (13)	0.95103 (4)	0.03528 (13)	
O1	0.0766 (5)	0.0993 (2)	1.2170 (5)	0.0495 (14)	
O2	0.1771 (6)	0.0886 (2)	0.6727 (6)	0.0517 (14)	
O3	0.3281 (5)	0.0295 (2)	0.7710 (6)	0.0507 (14)	
O4	−0.0904 (5)	0.08694 (18)	0.8702 (6)	0.0472 (13)	
O5	−0.2383 (5)	0.01441 (19)	0.8964 (6)	0.0458 (13)	
O6	0.0405 (5)	−0.02279 (18)	0.8341 (5)	0.0423 (12)	
N1	0.1482 (7)	0.1713 (2)	0.9431 (8)	0.0511 (16)	
N2	0.3590 (6)	0.1076 (2)	1.0623 (7)	0.0408 (14)	
C1	0.4668 (7)	0.0771 (3)	1.1138 (8)	0.0431 (19)	
H1A	0.4622	0.0380	1.1035	0.052*	
C2	0.5841 (8)	0.1002 (3)	1.1811 (9)	0.053 (2)	
H2A	0.6566	0.0772	1.2133	0.063*	
C3	0.5921 (9)	0.1571 (3)	1.1996 (10)	0.066 (3)	
H3A	0.6702	0.1733	1.2454	0.079*	
C4	0.4818 (8)	0.1915 (3)	1.1492 (10)	0.055 (2)	
C5	0.4823 (11)	0.2517 (4)	1.1624 (12)	0.084 (3)	
H5A	0.5565	0.2694	1.2124	0.101*	
C6	0.3792 (11)	0.2832 (4)	1.1049 (12)	0.086 (3)	
H6A	0.3830	0.3222	1.1162	0.103*	
C7	0.2628 (9)	0.2580 (3)	1.0260 (10)	0.064 (2)	
C8	0.1551 (10)	0.2892 (3)	0.9555 (11)	0.075 (3)	
H8A	0.1569	0.3285	0.9596	0.090*	
C9	0.0499 (11)	0.2627 (3)	0.8825 (12)	0.083 (3)	
H9A	−0.0214	0.2831	0.8352	0.100*	
C10	0.0503 (10)	0.2035 (3)	0.8793 (10)	0.067 (3)	
H10A	−0.0233	0.1855	0.8289	0.081*	
C11	0.2555 (8)	0.1984 (3)	1.0144 (9)	0.049 (2)	
C12	0.3674 (8)	0.1641 (3)	1.0785 (8)	0.0447 (19)	
C13	−0.0119 (7)	0.0640 (3)	1.2593 (8)	0.0391 (16)	
C14	−0.0864 (8)	0.0707 (3)	1.4136 (8)	0.055 (2)	
H14A	−0.1052	0.0342	1.4585	0.083*	
H14B	−0.1714	0.0903	1.3944	0.083*	
H14C	−0.0309	0.0921	1.4877	0.083*	
C15	0.2801 (8)	0.0583 (3)	0.6569 (9)	0.0431 (17)	
C16	0.3468 (9)	0.0548 (4)	0.4946 (9)	0.063 (2)	
H16A	0.4343	0.0736	0.4982	0.095*	
H16B	0.2889	0.0727	0.4156	0.095*	
H16C	0.3600	0.0159	0.4661	0.095*	
C17	−0.2064 (8)	0.0637 (3)	0.8529 (8)	0.0422 (17)	
C18	−0.3164 (8)	0.0977 (3)	0.7682 (9)	0.056 (2)	
H18A	−0.3995	0.0759	0.7617	0.084*	
H18B	−0.2862	0.1071	0.6615	0.084*	
H18C	−0.3334	0.1318	0.8275	0.084*	

### Atomic displacement parameters (Å^2^)

**Table:**

	*U*^11^	*U*^22^	*U*^33^	*U*^12^	*U*^13^	*U*^23^
Eu1	0.0401 (2)	0.02511 (17)	0.0406 (2)	−0.00140 (19)	−0.00452 (14)	−0.00032 (19)
O1	0.061 (4)	0.044 (3)	0.044 (3)	−0.015 (3)	0.004 (3)	−0.011 (2)
O2	0.061 (4)	0.045 (3)	0.049 (3)	0.014 (3)	−0.002 (3)	0.005 (2)
O3	0.046 (3)	0.056 (3)	0.049 (3)	0.011 (3)	−0.004 (3)	0.002 (3)
O4	0.041 (3)	0.036 (3)	0.064 (3)	−0.003 (2)	−0.009 (3)	0.007 (3)
O5	0.047 (3)	0.030 (3)	0.059 (3)	0.000 (2)	−0.014 (3)	0.008 (2)
O6	0.055 (3)	0.027 (3)	0.044 (3)	−0.005 (2)	−0.002 (2)	−0.011 (2)
N1	0.059 (4)	0.033 (3)	0.061 (4)	0.001 (3)	−0.008 (3)	0.001 (3)
N2	0.047 (4)	0.033 (3)	0.042 (3)	−0.006 (3)	0.001 (3)	−0.001 (3)
C1	0.039 (5)	0.035 (4)	0.055 (5)	0.002 (3)	−0.004 (4)	0.003 (3)
C2	0.048 (5)	0.049 (5)	0.062 (5)	−0.003 (4)	−0.012 (4)	−0.006 (4)
C3	0.064 (6)	0.055 (5)	0.077 (6)	−0.007 (5)	−0.019 (5)	−0.014 (5)
C4	0.051 (5)	0.039 (4)	0.075 (6)	−0.008 (4)	−0.007 (4)	−0.002 (4)
C5	0.092 (8)	0.046 (5)	0.114 (8)	−0.016 (5)	−0.034 (7)	−0.012 (6)
C6	0.106 (9)	0.033 (5)	0.118 (9)	−0.011 (5)	−0.021 (7)	−0.015 (5)
C7	0.075 (7)	0.035 (4)	0.081 (6)	−0.002 (4)	−0.008 (5)	−0.003 (4)
C8	0.096 (8)	0.031 (4)	0.099 (7)	0.010 (5)	−0.017 (6)	0.005 (5)
C9	0.101 (8)	0.038 (5)	0.110 (8)	0.014 (5)	−0.037 (7)	0.006 (5)
C10	0.071 (7)	0.045 (5)	0.086 (6)	−0.002 (4)	−0.027 (5)	0.006 (5)
C11	0.057 (5)	0.028 (4)	0.061 (5)	−0.009 (4)	0.003 (4)	−0.003 (4)
C12	0.055 (5)	0.031 (4)	0.048 (4)	−0.006 (4)	0.002 (4)	−0.001 (3)
C13	0.035 (4)	0.037 (4)	0.046 (4)	0.000 (4)	−0.001 (3)	0.000 (4)
C14	0.051 (5)	0.073 (6)	0.042 (4)	−0.006 (4)	0.009 (4)	−0.015 (4)
C15	0.040 (4)	0.031 (4)	0.059 (5)	−0.007 (4)	−0.007 (4)	−0.003 (4)
C16	0.065 (6)	0.074 (6)	0.052 (5)	−0.001 (5)	0.004 (4)	−0.001 (4)
C17	0.043 (4)	0.038 (4)	0.046 (4)	0.009 (4)	0.002 (3)	−0.001 (4)
C18	0.052 (6)	0.059 (5)	0.057 (5)	0.003 (4)	−0.007 (4)	0.011 (4)

### Geometric parameters (Å, °)

**Table:**

Eu1—O1	2.465 (5)	C4—C12	1.412 (9)
Eu1—O2	2.443 (5)	C4—C5	1.428 (10)
Eu1—O3	2.509 (5)	C5—C6	1.335 (11)
Eu1—O4	2.380 (5)	C5—H5A	0.9300
Eu1—O5^i^	2.387 (4)	C6—C7	1.434 (11)
Eu1—O6	2.372 (4)	C6—H6A	0.9300
Eu1—O6^i^	2.630 (5)	C7—C8	1.407 (10)
Eu1—N1	2.639 (6)	C7—C11	1.414 (9)
Eu1—N2	2.613 (6)	C8—C9	1.342 (11)
O1—C13	1.251 (8)	C8—H8A	0.9300
O2—C15	1.240 (8)	C9—C10	1.401 (10)
O3—C15	1.254 (8)	C9—H9A	0.9300
O4—C17	1.262 (8)	C10—H10A	0.9300
O5—C17	1.261 (8)	C11—C12	1.456 (10)
O5—Eu1^i^	2.387 (4)	C13—O6^i^	1.276 (7)
O6—C13^i^	1.276 (7)	C13—C14	1.484 (10)
O6—Eu1^i^	2.630 (5)	C14—H14A	0.9600
N1—C10	1.328 (9)	C14—H14B	0.9600
N1—C11	1.358 (8)	C14—H14C	0.9600
N2—C1	1.342 (8)	C15—C16	1.501 (11)
N2—C12	1.346 (8)	C16—H16A	0.9600
C1—C2	1.381 (9)	C16—H16B	0.9600
C1—H1A	0.9300	C16—H16C	0.9600
C2—C3	1.356 (10)	C17—C18	1.509 (9)
C2—H2A	0.9300	C18—H18A	0.9600
C3—C4	1.409 (11)	C18—H18B	0.9600
C3—H3A	0.9300	C18—H18C	0.9600
			
O6—Eu1—O4	75.50 (15)	C2—C3—H3A	120.1
O6—Eu1—O5^i^	76.58 (15)	C4—C3—H3A	120.1
O4—Eu1—O5^i^	136.96 (17)	C3—C4—C12	117.0 (7)
O6—Eu1—O2	84.76 (16)	C3—C4—C5	123.5 (7)
O4—Eu1—O2	79.46 (18)	C12—C4—C5	119.5 (7)
O5^i^—Eu1—O2	129.42 (18)	C6—C5—C4	121.8 (8)
O6—Eu1—O1	125.90 (17)	C6—C5—H5A	119.1
O4—Eu1—O1	86.14 (18)	C4—C5—H5A	119.1
O5^i^—Eu1—O1	84.40 (17)	C5—C6—C7	121.3 (8)
O2—Eu1—O1	141.47 (16)	C5—C6—H6A	119.3
O6—Eu1—O3	78.98 (17)	C7—C6—H6A	119.3
O4—Eu1—O3	126.90 (16)	C8—C7—C11	117.3 (8)
O5^i^—Eu1—O3	77.95 (17)	C8—C7—C6	123.7 (8)
O2—Eu1—O3	52.29 (16)	C11—C7—C6	119.0 (8)
O1—Eu1—O3	145.03 (16)	C9—C8—C7	120.4 (8)
O6—Eu1—N2	145.30 (17)	C9—C8—H8A	119.8
O4—Eu1—N2	138.49 (16)	C7—C8—H8A	119.8
O5^i^—Eu1—N2	77.60 (16)	C8—C9—C10	118.3 (8)
O2—Eu1—N2	94.15 (18)	C8—C9—H9A	120.9
O1—Eu1—N2	73.60 (18)	C10—C9—H9A	120.9
O3—Eu1—N2	73.25 (18)	N1—C10—C9	124.7 (8)
O6—Eu1—O6^i^	75.36 (17)	N1—C10—H10A	117.7
O4—Eu1—O6^i^	71.21 (16)	C9—C10—H10A	117.7
O5^i^—Eu1—O6^i^	70.45 (16)	N1—C11—C7	122.6 (7)
O2—Eu1—O6^i^	147.77 (16)	N1—C11—C12	117.8 (6)
O1—Eu1—O6^i^	50.54 (15)	C7—C11—C12	119.6 (7)
O3—Eu1—O6^i^	142.95 (15)	N2—C12—C4	123.0 (7)
N2—Eu1—O6^i^	116.62 (16)	N2—C12—C11	118.2 (6)
O6—Eu1—N1	146.45 (16)	C4—C12—C11	118.7 (6)
O4—Eu1—N1	76.66 (18)	O1—C13—O6^i^	119.3 (7)
O5^i^—Eu1—N1	136.95 (16)	O1—C13—C14	120.7 (6)
O2—Eu1—N1	72.03 (18)	O6^i^—C13—C14	120.0 (7)
O1—Eu1—N1	69.92 (18)	C13—C14—H14A	109.5
O3—Eu1—N1	103.7 (2)	C13—C14—H14B	109.5
N2—Eu1—N1	62.53 (18)	H14A—C14—H14B	109.5
O6^i^—Eu1—N1	112.49 (18)	C13—C14—H14C	109.5
C13—O1—Eu1	99.3 (4)	H14A—C14—H14C	109.5
C15—O2—Eu1	94.4 (4)	H14B—C14—H14C	109.5
C15—O3—Eu1	91.0 (5)	O2—C15—O3	122.2 (7)
C17—O4—Eu1	137.6 (4)	O2—C15—C16	118.7 (7)
C17—O5—Eu1^i^	137.0 (4)	O3—C15—C16	119.1 (7)
C13^i^—O6—Eu1	164.5 (5)	C15—C16—H16A	109.5
C13^i^—O6—Eu1^i^	90.8 (4)	C15—C16—H16B	109.5
Eu1—O6—Eu1^i^	104.64 (16)	H16A—C16—H16B	109.5
C10—N1—C11	116.8 (7)	C15—C16—H16C	109.5
C10—N1—Eu1	123.3 (5)	H16A—C16—H16C	109.5
C11—N1—Eu1	119.8 (4)	H16B—C16—H16C	109.5
C1—N2—C12	117.2 (6)	O4—C17—O5	126.3 (6)
C1—N2—Eu1	121.8 (4)	O4—C17—C18	116.8 (7)
C12—N2—Eu1	120.9 (5)	O5—C17—C18	116.9 (7)
N2—C1—C2	123.9 (7)	C17—C18—H18A	109.5
N2—C1—H1A	118.1	C17—C18—H18B	109.5
C2—C1—H1A	118.1	H18A—C18—H18B	109.5
C3—C2—C1	119.1 (7)	C17—C18—H18C	109.5
C3—C2—H2A	120.5	H18A—C18—H18C	109.5
C1—C2—H2A	120.5	H18B—C18—H18C	109.5
C2—C3—C4	119.9 (7)		

Symmetry codes: (i) −*x*, −*y*, −*z*+2.
